# Supplementary material for: Ramadan Fasting Improves Body Composition without Exacerbating Depression in Males with Diagnosed Major Depressive Disorders
Source: Nutrients. 2021 Aug 7;13(8):2718. doi: 10.3390/nu13082718 (PMC8398343; doi:10.3390/nu13082718)
Supplement: Supplementary file 1 [file nutrients-13-02718-s001.zip › nutrients-1233847-supplementary.pdf]

Table S1: Study parameters, normal range and interpretation

Parameter	Normal range and interpretation
Body mass (kg)	ND
Height (cm)	ND
BMI (kg/m2)	<18.5=Underweight<br>18.5-24.9=Normal or Healthy Weight<br>25.0-29.9=Overweight<br>≥30.0=Obese
%BFP (%)	2-5%=Essential fat<br>6-13%=Athletes<br>14-17%Fitness<br>18-25%=Average<br>>25%=Obese
%TBWP (%)	50%-70%=Ideal for men (middle age)
BSA (m2)	ND
LM (kg)	ND
FM (kg)	ND
SBP (mmHg)	<120=Normal<br>120-129=Elevated<br>130-139=High blood pressure (Stage I)<br>140-179=High blood pressure (Stage II)<br>>180=Hypertensive crisis
DBP (mmHg)	<80=Normal<br>81-84=Elevated<br>85-90=High blood pressure (I)<br>90-119=High blood pressure (I)<br>>120=Hypertensive crisis
LDL (mmol/L)	<2.6mmol/L=Optimal<br>2.6 to 3.4 mmol/L =Near optimal<br>3.5 to 4.1 mmol/L =Borderline high<br>4.2 to 4.9 mmol/L =High.
HDL (mmol/L)	>1.17 mmol/L=Acceptable<br>1.04-1.17 mmol/L=Borderline<br><1.04 mmol/L=Low Level
TG (mmol/L)	<1.7 mmol/L=Normal<br>1.8 to 2.2 mmol/L=Borderline high<br>2.3 to 5.6 mmol/L=High<br>≥5.7 mmol/L=Very high
TC (mmol/L)	<5.2 mmol/L=Desirable<br>5.2-6.2 mmol/L=Borderline high<br>>6.2 mmol/L=High
FBG (mmol/L)	<5.6 mmol/L=Normal<br>5.6 to 6.9 mmol/L=Prediabetes<br>>7 mmol/L=Diabetes
WC (cm)	ND
HC (cm)	ND

WHR (ratio)	<0.95=Low<br>0.96–1.0=Moderate<br>>1.0=High
MS z-score	Lower is better.
PHQ-9	1-4=Minimal depression<br>5-9=Mild depression<br>10-14=Moderate depression<br>15-19=Moderately severe depression<br>20-27=Severe depression

ND=not defined; BMI = body mass index; %BFP = body fat percentage; %TBWP = total body water percentage; BSA = body surface area; LM = lean mass; FM = fat mass; SBP = systolic blood pressure; DBP = diastolic blood pressure; LDL = low-density lipoprotein cholesterol; HDL = low-density lipoprotein cholesterol; TG = triglycerides; TC = total cholesterol; FBG = fasting blood glucose; WC = waist circumference; HC = hip circumference; WHR = waist to hip ratio; MS z-score = metabolic syndrome z-score; PHQ-9 = patient health questionnaire -9.
